# The need for speed in advanced non‐small cell lung cancer: A population kinetics assessment

**DOI:** 10.1002/cam4.4411

**Published:** 2021-11-11

**Authors:** David J. Stewart, Donna E. Maziak, Sara M. Moore, Stephanie Y. Brule, Marcio Gomes, Harman Sekhon, Carole Dennie, Bryan Lo, Michael Fung‐Kee‐Fung, John‐Peter Bradford, Martin Neil Reaume

**Affiliations:** ^1^ Department of Medicine University of Ottawa Ottawa Ontario Canada; ^2^ Department of Surgery University of Ottawa Ottawa Ontario Canada; ^3^ Department of Pathology University of Ottawa Ottawa Ontario Canada; ^4^ Department of Diagnostic Imaging University of Ottawa Ottawa Ontario Canada; ^5^ Department of Obstetrics and Gynecology University of Ottawa Ottawa Ontario Canada; ^6^ Life Saving Therapies Network Ottawa Ontario Canada

**Keywords:** non‐small cell lung cancer, overall survival, population kinetics, therapy delay

## Abstract

**Background:**

Systemic therapy prolongs overall survival (OS) in advanced non‐small cell lung cancer (NSCLC), but diagnostic tests, staging and molecular profiling take time, and this can delay therapy initiation. OS approximates first‐order kinetics.

**Methods:**

We used OS of chemo‐naive NSCLC patients on a placebo/best supportive care trial arm to estimate % of patients dying while awaiting therapy. We digitized survival curves from eight studies, calculated OS half‐life, then estimated the proportion surviving after different times of interest (*t*_n_) using the formula: $X\;=\;\exp\left(‐t_{n}*0\text{.693}/t_{1/2}\right)$, where EXP signifies exponential, * indicates multiplication, 0.693 is the natural log of 2, and *t*_1/2_ is the survival half‐life in weeks.

**Results:**

Across trials, the OS half‐life for placebo/best supportive care in previously untreated NSCLC was 19.5 weeks. Hence, based on calculations using the formula above, if therapy were delayed by 1, 2, 3, or 4 weeks then 4%, 7%, 10%, and 13% of all patients, respectively, would die while awaiting treatment. Others would become too sick to consider therapy even if still alive.

**Conclusions:**

This quantifies why rapid baseline testing and prompt therapy initiation are important in advanced NSCLC. It also illustrates why screening procedures for clinical trial inclusion must be faster. Otherwise, it is potentially hazardous for a patient to be considered for a trial due to risk of death or deterioration while awaiting eligibility assessment. It is also important to not delay initiation of systemic therapy for procedures that add relatively little value, such as radiotherapy for small, asymptomatic brain metastases.

## Abstract 2

In patients with metastatic non‐small cell lung cancer, it takes time for diagnostic and staging tests and for initiation of therapy. Based on exponential decay nonlinear regression analysis of overall survival curves from placebo or best supportive care arms of first line studies in metastatic disease, we calculated that an average of 4% of remaining patients will die during each week that passes without initiation of systemic therapy. It is important to initiate therapy as rapidly as possible.

## 1 INTRODUCTION

### 1.1 Systemic therapies in non‐small cell lung cancer

Many patients with metastatic non‐small cell lung cancer (NSCLC) derive benefit from systemic therapies such as chemotherapy, targeted agents, and immunotherapy. When compared to placebo or best supportive care, chemotherapy significantly prolongs overall survival (OS).^1^ While there is a common perception that it is of limited benefit in poor performance status patients, the relative improvement in OS is approximately the same in poor performance status patients as it is in good performance status patients.^1^ Chemotherapy is associated with improvement in cancer symptoms in more than 75% of good risk patients with longer OS but can also benefit high risk patients anticipated to have very short OS, with 55% of the high risk group experiencing symptomatic improvement.^2^

While chemotherapy is superior to no treatment in this population, patients whose tumors are found to have specific mutations or immune system biomarkers do better with targeted therapies or with immunotherapy than with chemotherapy.^3^, ^4^, ^5^ Good therapies are generally most effective when given as first line treatment. To choose the best first line therapy requires baseline diagnostic tests, staging, and molecular profiling, but patients are at risk of deteriorating rapidly and dying while awaiting completion of testing prior to therapy initiation. There can be long delays in referral of lung cancer patients for subspecialist care and in initiation of appropriate therapy, with several factors contributing to these delays.^6^

Initiation of systemic therapy can also be delayed if a patient is being assessed for possible inclusion in a clinical trial. In some cases, screening for a clinical trial can take weeks, particularly if a pre‐treatment research biopsy is required or if a biopsy must be assessed in a central lab prior to patient inclusion in the trial. Systemic therapy can also be delayed to permit prior completion of radiotherapy for brain metastases, and many clinical trials that permit patients with brain metastases require that the patient undergo cranial radiation and then demonstrate stability of their brain metastases for 4 weeks or more before the patient can be entered on the trial.

### 1.2 Implications of OS following first order kinetics

For most cancers, OS and progression‐free survival (PFS) approximate first order kinetics and can be fit by exponential decay nonlinear regression models.^7^, ^8^ This means that one can use a population kinetics approach to calculate OS and PFS half‐lives (*t*_1/2_: the time to death or progression of half of all remaining patients). OS and PFS half‐lives correlate very strongly with OS and PFS medians,^8^ but conceptualizing them as half‐lives has advantages.^8^, ^9^, ^10^ One advantage is that one may use the OS half‐life to easily calculate the proportion of remaining patients who will die per unit of future time, and this proportion will be roughly the same no matter where you start the clock if OS follows first‐order kinetics. For example, if the half‐life were 4 months, then half the patients would be dead by 4 months, half of the remaining patients (or three quarters of the original population) would be dead after another 4 months, and half the patients remaining at 8 months would die during the next 4‐month period so that about 12.5% of the original population would still be alive at 12 months.

We used population kinetics assessments to estimate % of NSCLC patients dying per week that therapy initiation is delayed. To do this, we assessed published NSCLC clinical trials with a placebo or best supportive arm and calculated the OS half‐life for patients who have not received any systemic therapy.

## 2 METHODS

### 2.1 Population kinetic assessment of advanced, untreated NSCLC

We conducted a PubMed search to identify all published clinical trials for advanced NSCLC that had a placebo/best supportive care arm. For our assessments, we then used the subgroup of these trials that involved previously untreated patients, that had ≥50 patients per arm and that had published OS Kaplan–Meier curves. As previously described,^8^, ^9^ we used the online application (https://apps.automeris.io/wpd/) to digitize these published OS curves. To estimate OS half‐life, we used GraphPad Prism‐7 (GraphPad Software) for 1‐phase and 2‐phase exponential decay nonlinear regression analysis (EDNLRA) of digitized curve data. In these calculations, we applied the constraints *Y*_0_ = 100 (since survival starts at 100%) and Plateau = 0 (since all patients would eventually die, if followed for a long enough time).^8^, ^9^ The use of Kaplan–Meier curves corrected for censored data. We excluded data from the terminal portion of the curve estimated to have <10 remaining patients, where each event would potentially cause relatively large curve changes.

Based on our prior experience,^8^, ^9^ we defined curves as fitting 2 phase EDNLRA models if both subpopulations accounted for at least 1% of the total population and if the half‐lives for the two potential subpopulations differed by ≥2 fold. In previous publications, we have illustrated examples of curves that fit 2 phase EDNLRA models.^7^, ^9^, ^11^ We have also previously published examples of how some individual EDNLRA curves compare to the corresponding Kaplan–Meier curves (Supplementary online figure 1 from Ref. [8]).

### 2.2 Estimation of proportion of patients surviving at specified times

As previously described for PFS curves,^9^ we then calculated the proportion of patients remaining alive after a time of interest, *t*_n_ (e.g. 1 week, 2 weeks, etc.), using the formula:

$$X\;=\;\exp\left(‐t_{n}*0\text{.693}/t_{1/2}\right),$$

where EXP signifies exponential, * indicates multiplication and *t*_1/2_ is the OS half‐life in weeks. The term 0.693 is the natural logarithm of 2. Another way of expressing this calculation would be:

$$x=2^{\left(‐t_{n}/t_{1/2}\right)}.$$

The percent dying by time *t*_n_ was calculated as 100 − (*x* * 100).

## 3 RESULTS

### 3.1 Proportion of untreated patients dying per week

Seven trials^12^, ^13^, ^14^, ^15^, ^16^, ^17^, ^18^ and a meta‐analysis^1^ fit the selection criteria described above. Across these trials, OS half‐lives by 1 phase decay models were 3.1–5.6 months (median, 4.5 months or 19.5 weeks). With an OS half‐life of 19.5 weeks, the proportions of remaining patients estimated to have died by 1, 2, 3, and 4 weeks from the start point were 4%, 7%, 10%, and 13%, respectively. This is in keeping with the rapid drop off in OS on most published Kaplan–Meier curves.

### 3.2 Proportion of good performance status patients dying if untreated

If we only considered trials in which the majority of patients were Eastern Cooperative Oncology Group Prformance Status 0–1, then OS half‐lives ranged from 3.7 to 5.6 months (median, 5.3 months or 23.0 weeks), and the proportion of patients estimated to have died by 1, 2, 3, and 4 weeks were 3%, 6%, 9%, and 11%, respectively.

### 3.3 Implications of OS 2 phase decay on exponential decay nonlinear regression analysis

In this analysis, five of eight OS curves fit 2 phase decay EDNLRA models (Table 1), suggesting two distinct subpopulations with differing survival rates. This is much higher (*p* = 0.001) than the proportion of OS curves fitting 2‐phase decay models in our earlier analyses involving patients receiving active treatment on both arms of a trial, where only 21 (11%) of 190 OS curves fit 2 phase decay models.^8^ This high probability of 2 phase decay is probably best explained by some of the patients being started on active therapy when tumor progression was detected, with shorter survival in the subpopulation never receiving any systemic therapy and longer survival in the subpopulation that eventually received systemic therapy. Assessment of individual patient data (to which we did not have access) would be required to test this assumption.

**TABLE 1:** Overall survival (OS) population kinetics analysis

Author	No. patients	PS^a^	1 phase decay model		2 phase decay model^c^			
			OS *t*_1/2_ ^b^ (CI)	*R* ^2^	% short^d^ (CI)	OS *t*_1/2_ short^e^ (CI)	OS *t*_1/2_ long^f^ (CI)	*R* ^2^
Ranson et al.^12^	78	0–1: 82% 2: 18%	5.6 (5.4–5.9)	0.97				
Rapp et al.^13^	53	0–1: 60% 2: 40%	3.7 (3.6–3.7)	0.99				
Roszkowski et al.^14^	70	0–1: 77% 2: 23%	5.3 (5.0–5.7)	0.94				
Goss et al.^15^	100	2: 62% 3: 38%	3.1 (3.0–3.3)	0.98	92 (88–96)	2.6 (2.4–2.9)	>100 (6.2–?)	0.98
Gridelli et al.^16^	78	0–1: 76% 2: 24%	5.5 (5.4–5.5)	0.99	7 (0.1–?)	2.0 (0.2–?)	5.8 (5.4–?)	0.99
Lee et al.^17^	320	0–1: 16% 2: 56% 3: 28%	3.9 (3.8–4.0)	0.99	89 (80–94)	3.2 (2.9–3.4)	20.3 (11.0–?)	0.99
Woods et al.^18^	91	0–1: 73% 2–3: 27%	4.5 (4.4–4.7)	0.99	33 (8–94)	2.4 (1.0–4.0)	6.1 (5.0–?)	0.99
NSCLC Collaborative Group^1^	362	?	4.4 (4.3–4.5)	0.99	96 (90–97)	3.9 (3.7–?)	>100 (17.8–?)	0.99

Abbreviations: CI, 95% confidence intervals; NSCLC, non‐small cell lung cancer.

^a^ PS: Eastern Cooperative Oncology Group Performance Status.

^b^ OS *t*_1/2_: overall survival half‐life (months) by an exponential decay nonlinear regression analysis 1‐phase decay model.

^c^ If no values are listed, data did not fit an exponential decay nonlinear regression analysis 2‐phase decay model per our definition.

^d^ % of entire population belonging to subpopulation with shorter OS.

^e^ OS half‐life (months) for subpopulation with shorter OS.

^f^ OS half‐life (months) for subpopulation with longer OS. Confidence intervals are very wide or undefinable since length of follow up was too short.

The subpopulation with shorter survival in trials with 2 phase decay in the current analysis accounted for 7%–96% of the entire study population (median across trials of 89%), and the subpopulation with shorter OS had an OS half‐life of 2.0–3.9 months (median across trials of 2.6 months, or 11.3 weeks) (Table 1). The proportion of this potentially large subpopulation of patients that were estimated to have died by 1, 2, 3, and 4 weeks was 6%, 12%, 17%, and 22%, respectively.

## 4 DISCUSSION

### 4.1 Implications of treatment delays in advanced NSCLC

This population kinetics analysis provides an estimate of the proportion of patients with advanced NSCLC who die per week of delay in initiation of systemic therapy and illustrates that even very short delays in initiation of therapy can translate into worsened survival. This calculation in NSCLC patients with advanced disease mirrors the situation in early stage NSCLC: in early stage, NSCLC short delays in initiation of therapy also worsen outcome.^19^ Patients with metastatic NSCLC may die rapidly if not treated, even if performance status is initially good. Many others will deteriorate so rapidly that they can no longer be considered for systemic therapy. For patients with advanced NSCLC, poor performance status is the leading reason for failure to receive systemic therapy at our center,^20^ and rapid deterioration and death may be the major reason that fewer than 25% of patients with advanced NSCLC in the province of Ontario, Canada ever receive systemic therapy,^21^ despite it being fully funded by the government.

In addition to performance status, other clinical factors could potentially impact the probability that an individual patient could die while awaiting initiation of therapy. For example, patients with high tumor bulk generally would be more likely to die rapidly than would patients with lower tumor bulk. Patients with stage IV disease generally would be more likely to die rapidly than patients with incurable stage IIIB disease. The papers included in our analysis had insufficient published information to permit us to quantify the effects of these factors. However, while sicker patients are the most likely to die rapidly if not treated, patients can go from being relatively “healthy” to being sick in a very short period of time. Consequently, speed is important for all patients.

### 4.2 Use of a population kinetic approach versus other methodology options

Direct inspection of NSCLC OS Kaplan–Meier curves supports the conclusion that patients die rapidly if not treated. While one might calculate the proportion of remaining patients dying per unit time directly from the Kaplan–Meier curve or through other statistical approaches, determination of an OS half‐life facilitates this calculation. In addition if one uses log‐linear plots of OS versus time and subjects the data to population kinetic exponential decay nonlinear regression analysis, one may readily see if the rate of death slows over time (e.g., due to presence of two distinct subpopulations with different OS half‐lives) or if it accelerates over time (e.g., due to discontinuation of a therapy that was slowing tumor growth).^8^, ^11^ Using log‐linear relationships is already used in standard statistical calculations (e.g., in determination of hazard ratios), and population kinetic approaches to OS and PFS data analysis can also be useful in a variety of other ways.^7^, ^8^, ^9^, ^10^, ^11^

While concern might be raised about using digitized data from published Kaplan–Meier curves, our experience suggests that this approach is highly reliable. For example, PFS and OS half‐lives derived from digitized curve data correlate very highly with their respective median values, with *R* ^2^ ≥ 0.96, and gain in PFS half‐life is a better predictor of OS gain than is gain in median PFS or PFS hazard ratio.^8^

### 4.3 **Are the placebo/best supportive care (BSC) data** **representative** **?**

One potential limitation of our assessment is the fact that the data used were from relatively old trials, published between 1988 and 2012. Hence, current untreated patients might possibly do better than the patients on the placebo/BSC arms of the trials assessed. However, we are unaware of any data to support this. Furthermore, even if the OS half‐life in untreated patients was 6 months (instead of the 4.5 months calculated), this would still mean that almost 3% of remaining patients would die each week that therapy initiation is delayed, and 2% would die each week if OS half‐life was 8 months.

### 4.4 Importance of rapid diagnostic assessment

While systemic therapy cannot cure advanced NSCLC, multiple trials have demonstrated that even older therapies do make a difference, with both prolongation of life expectancy^1^, ^12^, ^13^, ^14^, ^16^ and improvement in quality of life and/or cancer‐related symptoms compared to placebo/best supportive care.^12^, ^14^, ^16^ There are several new therapies that are substantially better in selected populations than these effective old therapies.^3^, ^4^, ^5^ However, it is essential to choose the right therapy. Overall, baseline testing to establish the diagnosis, stage and molecular profile of advanced NSCLC is highly important, but this testing takes time. Our estimates here drive home how essential it is to make this testing happen as rapidly as possible.

### 4.5 Importance of rapid screening for clinical trial inclusion

There are also other things that can delay therapy initiation. For example, if a patient is being considered for inclusion in a clinical trial, it may take weeks longer to initiate treatment than if the patient goes straight to standard therapy, particularly if a research biopsy is required.^22^ The data we present here illustrate why it is vitally important that we address these delays so that we can continue to make progress through clinical research without jeopardizing patients being considered for inclusion in a trial.

### 4.6 Potential impact of brain metastases on rapidity of initiation of systemic therapies

Of particular concern in clinical research is the requirement in some studies that patients undergo cranial radiation for brain metastases and then demonstrate stability for a number of weeks before being considered for study‐related systemic therapies. Even good performance status patients are at risk of deteriorating or dying from uncontrolled extracranial tumor during this mandatory wait period.

A strong case can be made for including patients with untreated asymptomatic brain metastases on clinical trials without requiring prior cranial radiation or demonstration of stability of the brain metastases.^23^, ^24^, ^25^ Contrary to a pervasive bias, brain metastases are not associated with worse OS than are metastases in a variety of other extrathoracic sites such as liver, bone or adrenal gland,^26^, ^27^, ^28^ blood–brain barrier disruption within brain metastases means that systemic agents gain ready access to these tumor deposits even if concentrations in the normal central nervous system are low,^25^ and efficacy of systemic therapies in untreated NSCLC brain metastases is not substantially different from efficacy in other disease sites.^24^ In keeping with this, brain metastases are not considered differently than other extrathoracic metastases in NSCLC Tumor Node Metastasis staging algorithms.^29^

Even in standard practice, careful consideration should be given to whether any local therapy (such as cranial radiation for asymptomatic brain metastases) should be given prior to initiation of systemic therapy. There is an increasing body of evidence that systemic therapy may prove effective against NSCLC brain metastases,^23^, ^24^ and rapid initiation of this systemic therapy is highly important in optimizing patient outcome.

### 4.7 Other reasons for delaying therapy initiation

There are also other reasons why therapy initiation might be delayed. For example, therapy might be delayed in an asymptomatic patient, where therapy toxicity might worsen quality of life. In such patients, there is no right answer as to when therapy should be initiated. For symptomatic patients, there is no question that systemic therapies may improve cancer symptoms and some aspects of quality of life.^2^, ^12^, ^14^, ^16^ However, some individual patients may enjoy several months prior to onset of cancer symptoms. In keeping with the well‐established impact of performance status on life expectancy, one would expect a relatively low proportion of good performance status patients to die rapidly from their cancer. On the other hand, some can go from being asymptomatic to being highly symptomatic and seriously ill over the course of just a very few weeks.

### 4.8 Conclusions

Several factors can delay initiation of systemic therapy,^6^ but a high price may potentially be paid for these delays. However, long delays are not inevitable. At our center, we undertook a Lung Cancer Transformation exercise that reduced the time from initial referral for diagnostic testing to initiation of therapy by 48% by optimizing a Lung Diagnostic Assessment Program that rapidly screens submitted consults, orders all required tests (based on initial information submitted), and triages patients to discussion at multidisciplinary rounds and/or prompt appointments with the most relevant providers.^30^ There are other opportunities for still further progress and it is essential that we seize these opportunities.

## CONFLICT OF INTERESTS

D. Stewart has received personal fees from Roche, Boehringer Ingelheim (BI), Merck, and AstraZeneca (AZ). He also owns a minor interest on US Patent no. 9.675.663 (test to predict response to TUSC2/FUS1 gene therapy). S. Brule has received personal fees from BMS, AZ, Takei, Bayer, BI, Merck, Roche. M. Gomes has received personal fees from Roche, AZ, BI, Merck and Amgen and grant support from AZ, Roche, BI, Merck, BMS, Pfizer, Eli Lilly (EL). M. Fung‐Kee‐Fung has received personal fees from AZ, Roche, Merck. The Ottawa Hospital has received clinical trials support from several different pharmaceutical companies. D. Maziak, S. Moore, B. Lo, H. Sekhon, J.P. Bradford, C. Dennie, M.N. Reaume have nothing to disclose.
